# Multiple traction band—assisted endoscopic submucosal dissection of a giant colorectal lipoma after unsuccessful unroofing

**DOI:** 10.1016/j.vgie.2026.03.003

**Published:** 2026-03-27

**Authors:** Hiroshi Yamazaki, Yohei Minato, Koichi Furuta, Shinya Nagae, Nao Takeuchi, Ryoju Negishi, Hideki Chiba, Ken Ohata

**Affiliations:** 1 Department of Gastrointestinal Endoscopy, NTT Medical Center Tokyo, Tokyo, Japan; 2 Department of Gastroenterology, Itabashi Chuo Medical Center, Tokyo, Japan; 3 Department of Gastroenterology, Omori Red Cross Hospital, Tokyo, Japan

## Abstract

**Background and Aims:**

Large colorectal lipomas can cause symptoms such as abdominal pain, bleeding, and intussusception. Despite increasing reports of endoscopic treatments, endoscopic resection of large lipomas remains technically challenging. Here, we report a case of successful endoscopic submucosal dissection (ESD) using multiple traction bands for a large lipoma after failed unroofing.

**Methods:**

A 55-year-old woman with chronic abdominal pain had a 70-mm submucosal tumor in the ascending colon, confirmed to be a lipoma. Initial unroofing failed because of poor electrical conductivity of the lipoma. Two months later, with the patient having persistent symptoms and a remaining 50-mm lipoma, ESD was performed. Multiple traction bands were clipped between the lipoma and the contralateral wall to lift the tumor and improve visualization.

**Results:**

ESD was successfully performed using multiple traction devices applied sequentially to maintain tension and visibility during dissection. The lesion was completely removed en bloc, and the defect was closed using the clip-with-line pulley–securing technique. Histology confirmed a benign lipoma. The patient remained symptom free with no recurrence at 1-year follow-up.

**Conclusions:**

Multiple traction–assisted ESD is a safe and effective approach for large colorectal lipomas, enabling minimally invasive resection while reducing the risk of perforation and recurrence.

## Introduction

Large intestinal lipomas can cause abdominal symptoms such as abdominal pain, bleeding, and intussusception,^1^ often necessitating surgical resection.^1^ Despite increasing reports of endoscopic treatments,^2^, ^3^, ^4^ endoscopic resection of large lipomas remains technically challenging. Here, we present a case of a large colorectal lipoma resected by endoscopic submucosal dissection (ESD) assisted with multiple traction bands after an unsuccessful initial attempt at unroofing.

## Case

A 55-year-old woman with chronic abdominal pain underwent colonoscopy, revealing a 70-mm soft reddish submucosal tumor in the ascending colon. Abdominal CT scan showed features suggestive of colorectal lipoma. Initial attempts at unroofing were unsuccessful because the poor electrical conductivity of the fat tissue prevented effective snaring. Multiple small resections of the mucosa were performed until spontaneous protrusion of the yellowish fat tissue was observed. Two months later, a 50-mm lipoma persisted, prompting resection by ESD to prevent symptoms and potential intussusception.

## Procedure and outcome

**Figure 1:**
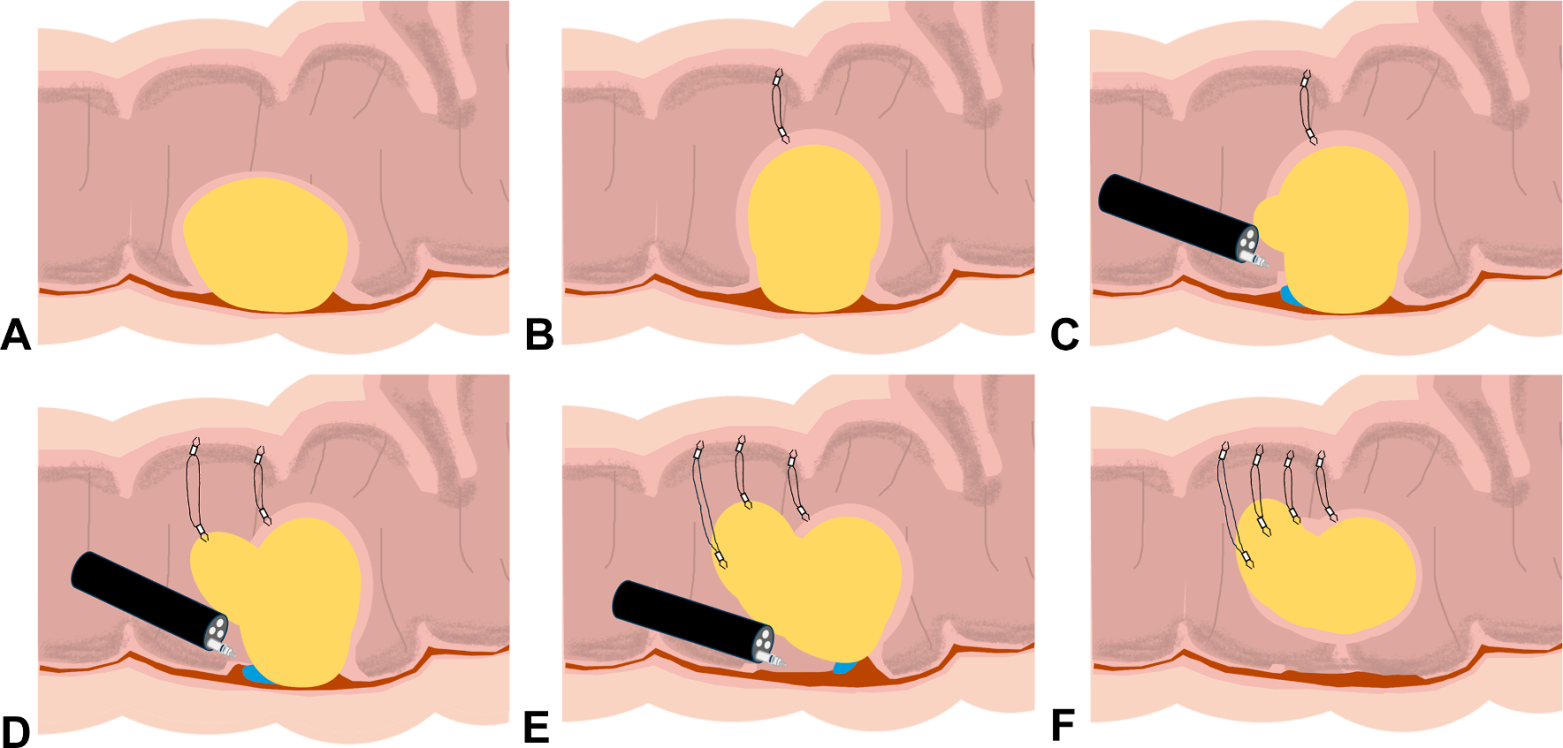
Schema of the multiple traction–assisted colorectal endoscopic submucosal dissection. **A,** The lipoma is embedded in the submucosal layer. **B,** Initial traction is applied to lift the lipoma. **C,** Incision and dissection are initiated from the proximal side. **D** and **E,** Additional tractions are applied appropriately to maintain clear view of the dissection plane. **F,** The lipoma is resected en bloc.

**Figure 2:**
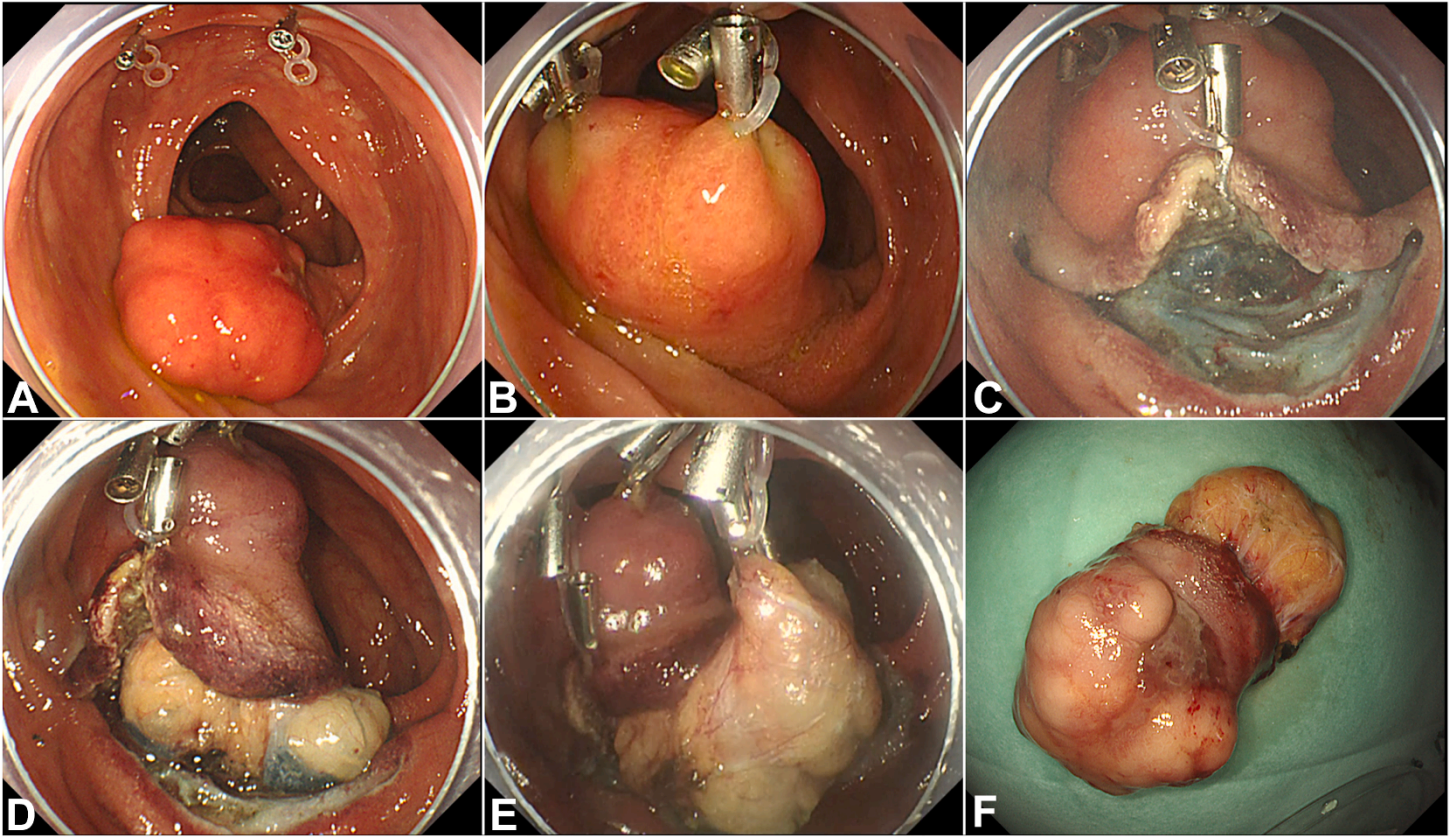
Endoscopic images of the multiple traction-assisted colorectal endoscopic submucosal dissection. **A,** Lipoma in the ascending colon. Traction bands are clipped onto the colorectal wall. **B,** Initial traction bands are applied to lift the lipoma away from the muscular layer. **C,** Incision is performed from the proximal side, and a traction band is applied to open the dissection plane. **D** and **E,** Additional tractions are applied appropriately as the dissection proceeded. **F,** The lipoma is resected en bloc.

Based on the CT findings indicating that the large lipoma was compressed against the luminal wall and likely embedded deep within the submucosal layer, it was anticipated that the ESD would be more challenging. To overcome this problem, 2 elastic traction devices (SureClip traction band, Micro-Tech, Nanjing, China) were clipped onto the colorectal wall on the counter-gravity side, with the other end of each band clipped onto the lipoma. The pulling force of the traction bands lifted the lipoma away from the muscle layer, improving visibility of the base. After the mucosal incision of the proximal side using a needle-type knife (Tech Knife, Micro-Tech), an additional traction band was applied to the dissected mucosa to elevate the lesion and open the dissection plane. Because the dissection proceeded, further traction devices were applied appropriately to maintain separation of the lipoma from the muscle layer and ensure a clear view of the submucosal layer (Figs. 1 and 2). After a complete resection of the lipoma, the defect was closed using the clip-with-line pulley–securing technique.^5^ Histologic analysis confirmed a benign lipoma. At 1-year follow-up, the patient remained asymptomatic with no endoscopic signs of recurrence (Video 1, available online at www.videogie.org).

## Discussion

Despite the generally safe nature of unroofing,^6^^,^^7^ treatment failure can occur, particularly with large lesions.^8^ In this case, electrocautery was ineffective because of the insulating properties of fat tissue,^4^ and piecemeal unroofing had to be performed. However, because the lipoma persisted on follow-up, full resection was warranted.

ESD was preferred over EMR because EMR was considered high risk in view of past reports of perforation.^6^ Although ESD demands advanced endoscopic skills, it has the advantage of en bloc resection,^6^ eliminating the risk of residual tumor that could lead to recurrent symptoms.

Most lipomas originate from the submucosal layer, and this large lipoma, exceeding 50 mm, was predicted to be embedded in the deeper layer of the submucosa, which would make it difficult to delineate the boundaries between the lipoma and the muscle layer. Traction bands were applied to lift the lipoma away from the muscle layer, improving visualization and facilitating the procedure. However, because the dissection proceeded, the dissection plane became compressed by the mass itself. Additional tractions sequentially pulled the lipoma away from the muscle layer and maintained a clear view of the dissection plane, decreasing the risk of perforation.

To our knowledge, this is the first case report demonstrating the resection of a large lipoma using multiple traction bands. The method is straightforward, involving only clips and bands, and has proven effective, with no adverse events or recurrence observed during follow-up.

## Conclusion

Resection of large ileal lipoma using multiple tractions can facilitate the procedure and allow more lesions to be resected with minimally invasive and organ-preserving endoscopic procedures.

## Disclosure

All authors disclosed no financial relationships.

## Supplementary data

Video 1 Multiple traction band–assissted endoscopic submucosal dissection of a giant colorectal lipoma.
